# Brain structural abnormalities in six major psychiatric disorders: shared variation and network perspectives

**DOI:** 10.12688/f1000research.51475.1

**Published:** 2021-05-07

**Authors:** Euclides José de Mendonça Filho, Márcio Bonesso Alves, Patricia Pelufo Silveira

**Affiliations:** 1 Department of Psychiatry, McGill University, Montreal, Quebec, H3A 1A1, Canada; 2 Ludmer Centre for Neuroinformatics & Mental Health, Douglas Mental Health University Institute, Montreal, Quebec, H4H 1R3, Canada

**Keywords:** Cross-disorder, ENIGMA, Psychiatric disorders, Structural MRI, Principal Component Analysis, Network Analysis

## Abstract

Common brain abnormalities are a possible explanation for comorbidities in psychiatric disorders. Challenges in understanding these conditions are likely due to the paucity of studies able to analyze the extent and regional distribution of shared morphometric abnormalities between disorders. Recently, Opeal *et al.* presented an elegant rationale to investigate shared and specific morphometric measures of cortical thickness and subcortical gray matter volume between healthy individuals and subjects across six major psychiatric disorders. Although their approach has the potential to systematically portrait shared brain alterations, the chosen principal component analysis solution may not address the central question of the observed shared versus specific brain alterations due to misspecification of the number of components. Given how this misspecification can lead to different conclusions, we reanalyzed Opel *et al. *data to thoroughly determine the number of factors to be considered, explore the alternative solution, and visualize the patterns of shared brain matter correlations using network analysis. Our approach suggests that a unidimensional solution was appropriate in this situation. The unidimensional solution indicated that brain alterations in autism spectrum disorder (ASD) had a significant negative component loading, suggesting that brain abnormalities found in ASD carry more similarities with major depressive disorder (MDD), bipolar disorder (BD), schizophrenia (SCZ), and obsessive-compulsive disorder (OCD) than demonstrated by the original work. Network analysis indicated that SCZ had the highest strength, BD the highest closeness, and BD and MDD had the highest betweenness in the network. This work highlights how different component solutions can lead to different conclusions, with important implications for the understanding of overlapped patterns of symptoms among six major psychiatric diseases. The network approach is complementary in indicating central markers of specific psychopathology domains. Investigations using shared-variation and network perspectives are promising for the study of pathophysiological patterns of common brain alterations.

## Introduction

Challenges to the understanding of heterogeneity and comorbidity of psychiatric disorders have long been acknowledged in medicine. The National Institute of Mental Health’s Research Domain Criteria (RDoC) initiative acknowledges that common brain abnormalities can potentially explain psychiatry comorbidities, ^1^ but few studies were able to systematically investigate the extent and regional distribution of shared morphometric abnormalities between disorders.

Using published meta- and mega-analyses of the Enhancing Neuro Imaging Genetics Through Meta-Analysis (ENIGMA) consortium, Opel *et al.* ^2^ present an elegant rationale to investigate shared and specific morphometric measures of cortical thickness and subcortical gray matter volume between typical control individuals and subjects with six of the major psychiatric disorders (major depressive disorder [MDD], bipolar disorder [BD], schizophrenia [SCZ], obsessive-compulsive disorder [OCD], attention-deficit/hyperactivity disorder [ADHD], and autism spectrum disorder [ASD]). To address whether brain-structural alterations related to these disorders loaded onto latent variables, shared brain abnormalities among them were examined using principal component analysis (PCA) across all cortical and subcortical regions. Then component scores were compared with the empirical regional effect sizes, allowing the definition of regions in each disorder that were better predicted by a shared variance component. The authors retained three principal components across disorders with a solution defined by MDD, BD, SCZ, and OCD loading on the first component, ADHD on the second, and ASD on the third component, leading to the conclusion that MDD, BD, SCZ, and OCD shared neuro-abnormality patterns, whereas ASD and ADHD exhibited disease-specific alterations.

Although the rationale used to investigate shared and specific morphometric measures of brain matter consists of an opportune strategy for improving the understanding of the pathophysiologic mechanisms of psychiatric disorders, ^3^ the number of components retained in the analysis, in this case, may not address the central question of the observed shared versus specific brain alterations. The three-factor solution parcels out the weaker residual correlation into minor components that might be of theoretical importance. We recognize that it is often a challenge to define the appropriate number of factors for data reduction, but common recommendations assume that retaining components with eigenvalues >1.0 usually indicates an excessive number of components. Moreover, the consideration of weak or poorly identified factors (i.e., components defined by only one or two variables) is an indication that the number of factors extracted should be reconsidered. ^4^

The over-extraction and under-extraction of factors retained in data reduction can have deleterious effects on the results. ^5^ Given the importance of the number of factors in data reduction and how different component solutions can lead to different conclusions, we leveraged the cross-disease effect sizes reported in Opel *et al.* ^2^ to thoroughly determine the number of factors to be considered, explore the appropriate solution, and visualize the patterns of shared brain matter correlations using network analysis.

## Methods

We reanalyzed the underlying structure of 41 regional measures of cortical thickness and subcortical volumes across the six psychiatric disorders compiled by Opel *et al.* ^2^ The data consisted of effect sizes obtained by contrasting healthy controls (N = 33,146) and patients (N = 19,578) of published structural neuroimaging mega- and meta-analyses of the ENIGMA consortium in the years 2016 to 2019. ^2^ The data selection criteria included the availability of effect sizes of psychiatric disorders for all 34 cortical brain regions based on the Desikan-Killiany atlas automated labeling system, ^6^ and 7 subcortical regions included in the standardized probabilistic information, modeled using the Markov random fields imaging pipeline ^7^ applied by the ENIGMA consortium. This criteria identified 11 studies of six psychiatric disorders: MDD (N = 2), BD (N = 2), SCZ (N = 2), OCD (N = 2), ADHD (N = 2), and ASD (N = 1). The effect size measures consisted of Cohen’s *d* mean differences in each cortical or subcortical region after age, sex, scanner adjustment, and in case of subcortical volume, total intracranial volume. ^2^

### Data source and preparation

The object of analysis is a dataset comprised of the effect size estimates for six psychiatric disorders and 41 regions of interest – hippocampus, amygdala, thalamus, accumbens, caudate, putamen, pallidum, isthmus cingulate cortex, posterior cingulate cortex, rostral anterior cingulate cortex, caudal anterior cingulate cortex, lateral orbit frontal cortex, pars opercularis of inferior frontal gyrus, rostral middle frontal gyrus, superior frontal gyrus, medial orbital frontal cortex, pars orbitalis of inferior frontal gyrus, pars triangularis of inferior frontal gyrus, caudal middle frontal gyrus, precentral gyrus, frontal pole, paracentral lobule, insula, lateral occipital cortex, lingual gyrus, cuneus, pericalcarine cortex, inferior parietal cortex, supramarginal gyrus, precuneus, superior parietal, postcentral gyrus fusiform gyrus, middle temporal gyrus, inferior temporal gyrus, banks superior temporal sulcus, superior temporal gyrus, parahippocampal gyrus, transverse temporal gyrus, entorhinal cortex, and temporal pole. This dataset was obtained by copying the effect sizes estimates reported by Opel *et al* ^2^ on Supplementary Table S2 of their manuscript. ^2^ The full manuscript was accessed in https://doi.org/10.1016/j.biopsych.2020.04.027 via the McGill Library Portal ( https://www.mcgill.ca/library/) on September 19, 2020. The data extracted from Opel’s manuscript ^2^ Supplementary Table S2 was prepared for statistical manipulation in the SPSS (V.21, IBM Corp., Armonk, NY) statistical environment and is available upon request.

### Data analysis

The number of principal components retained was determined using the scree plot criteria and Horn’s parallel analysis. ^8^ The scree plot shows how much variation each component captures from the data and allows to determine the inflection point in the data where additional components are unnecessary. The number of data points above the inflection is usually the number of components to retain. Horn’s parallel analysis compares the eigenvalues randomly generated from the data using Monte-Carlo simulation with the original data. The number of components retained consists of the original eigenvalues that are higher than the simulated eigenvalues. ^8^ We also verified the number of components in the cross-disease correlation matrix with eigenvalues greater than 1.0, although this procedure is considered one of the least accurate methods for selecting the number of components. ^5^ At this stage, we used the functions implemented by the *psych* package ^9^ from the R statistical language (V. 4.0.2). At a second stage, we used the SPSS software to conduct exploratory factor analysis. The principal components algorithm was used for dimensional extraction, and component scores obtained using the regression method. ^9^

To investigate specific patterns of residual correlations between the psychiatric disorders, we fitted a Gaussian graphical model with a Least Absolute Shrinkage and Selection Operator ( *gLASSO*) ^10^ to the data using the *qgraph* ^11^ R package. This procedure yields parsimonious partial-correlations of the brain alterations for each pair of psychiatric diseases here represented as nodes. Edges between diseases indicate a regularized partial correlation, after conditioning on all other diseases in the dataset. To assess the importance of nodes in the network, we computed the following centrality measures: strength, a measure of how well a node is directly connected to other diseases, closeness, how well a node is indirectly connected to other diseases, and betweenness, quantification of how important a node is in the average path between two other diseases.

## Results

**Figure 1.**
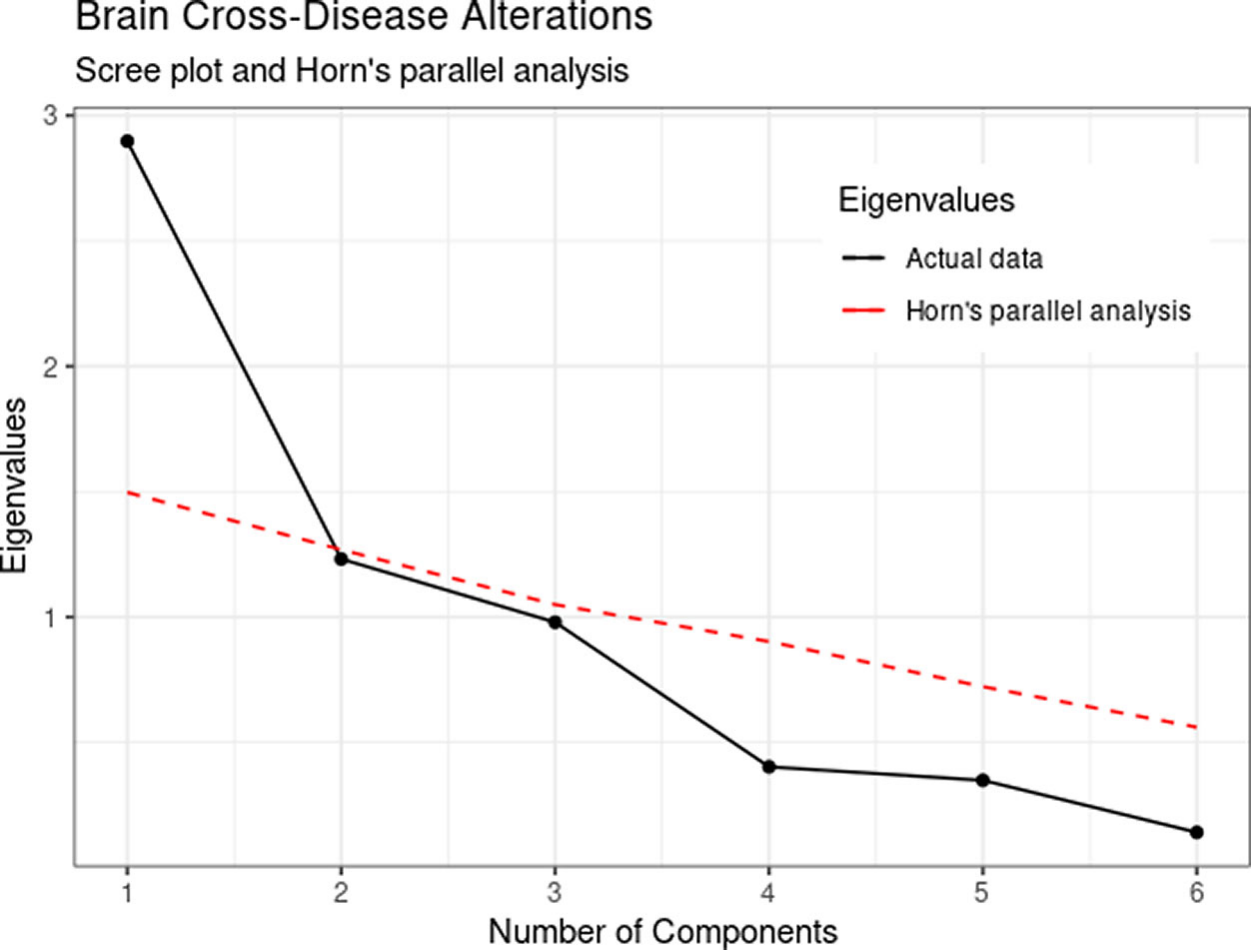
Eigenvalues scree plot and Horn’s parallel analysis of brain structural effect size alteration in six psychiatric disorders.

Inspection of the scree plot and Horn’s parallel analysis indicated the consideration of one component instead of the original three-component solution. Figure 1 shows a steeper decrease from the first to the second eigenvalues, followed by a flatter pattern for the remaining components. In addition, only the first actual eigenvalue was higher than the resampled eigenvalues (depicted in red) suggesting a unidimensional solution. It can be noticed that the third component obtained an eigenvalue of .98, violating the liberal eigenvalues greater than the 1.0 cut-off. Therefore, we opted to retain one component for subsequent analysis.

**Figure 2.**
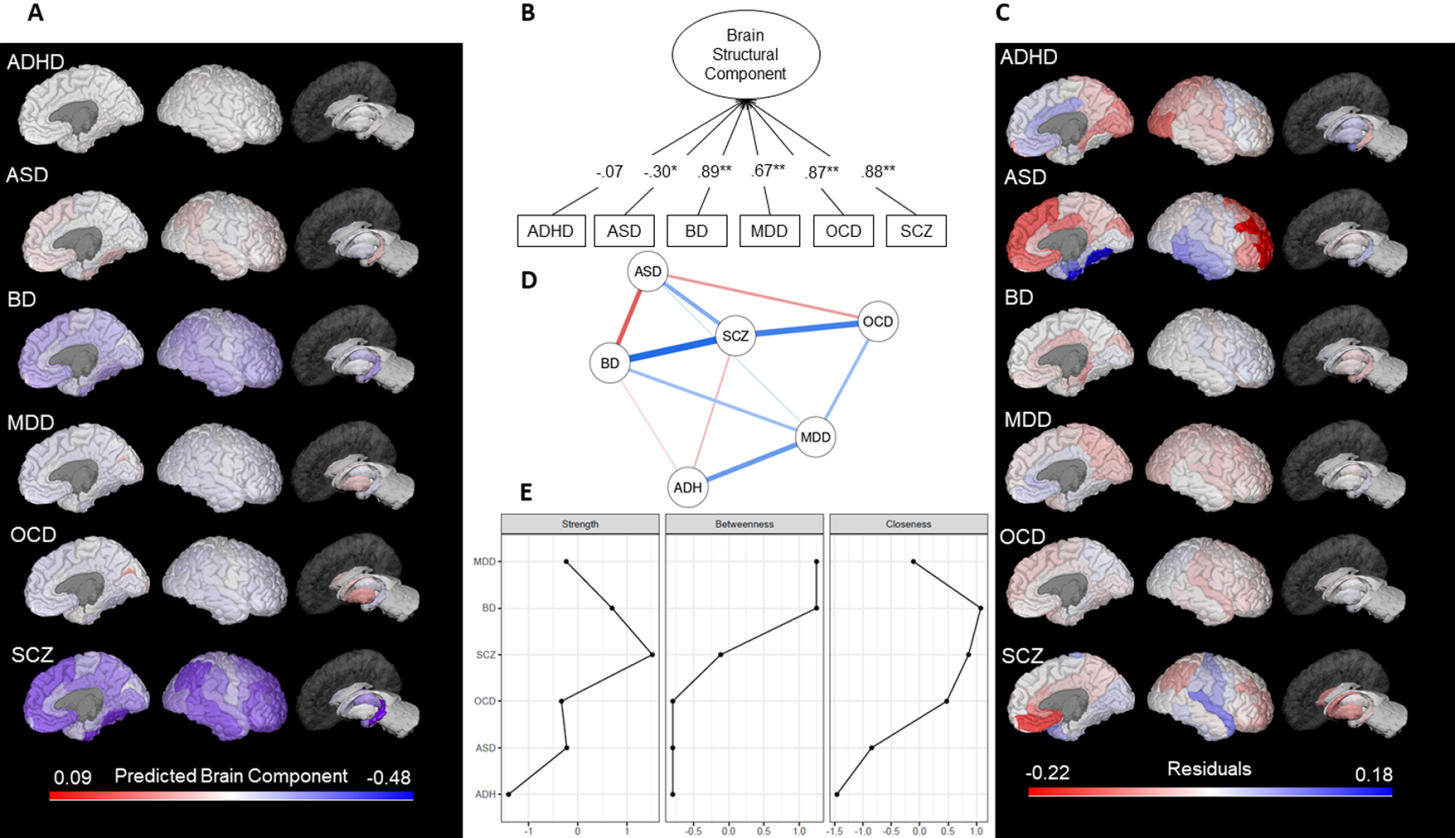
Principal component and network analysis of the effect sizes of brain structural alterations in six psychiatric disorders. (A) Predicted component scores mapped onto brain regions; (B) Path diagram of the principal component unidimensional solution; (C) Residuals from the regional effect sizes accounting for the predicted shared principal component. As in Opel *et al.*, ^2^ the absolute size of residuals encompasses the degree of representation through the shared unidimensional component. Lower (negative) residuals represent underestimation and higher (positive) residuals represent overestimation based on the brain-regional shared variance. (D) *gLASSO* correlations network of brain structural alterations in the six psychiatric disorders. Edges represent parsimonious partial-correlations between psychiatric disorders. A stronger correlation (positive = blue; negative = red) results in a thicker and darker edge. (E) Network centrality measures. ADHD, attention-deficit/hyperactivity disorder; ASD, autism spectrum disorder; BD, bipolar disorder; MDD, major depressive disorder; OCD, obsessive-compulsive disorder; SCZ, schizophrenia. All effect sizes used in these results are taken from Opel *et al.* ^2^ * *p* < 0.05; ** *p* < 0.001.

The unidimensional solution indicated that the effect size of the differences in brain structure between ASD patients and controls shared a significant negative component loading (λ = -0.30, *p* = 0.04) with the brain abnormalities of the remaining five diseases ( Figure 2). While in the original analysis SCZ had the highest shared correlation (indicated by the highest component loading) of the four conditions (MDD, BD, SCZ, and OCD), the unidimensional solution showed that the BD (λ = 0.89, *p* < 0.001) followed by SCZ (λ = 0.88, *p* < 0.001), and OCD (λ = 0.87, *p* < 0.001) had the highest shared correlation with all six disorders ( Figure 2B), illustrating how different dimensional reduction solutions are implied in different patterns of covariance. Similar to the original report, ADHD did not load into the PCA, indicating low shared brain abnormalities with the other disorders.

We also calculated a regional component score (M = 0.0, SD = 1.0) to identify which brain areas were more affected in a cross-disease manner. The hippocampus (-1.64) and the fusiform gyrus (-1.40) exhibited a more prominent shared reduction, whereas the pallidum (2.94) and putamen (2.09) showed a stronger shared increase. In order to explore shared- and disorder-specific morphometric abnormalities, we computed regional effect-sizes residuals from the component score. In contrast to the original work, this allowed the inclusion of ASD and ADHD in the analysis, although ADHD was not further explored due to its non-significant component loading. Regional specificities for MDD, BD, SCZ, and OCD were similar to Opel *et al.* ^2^ results ( Figure 2C). ASD showed large residuals especially for the rostral middle (residual [res] = .18) and superior frontal gyrus (res = .15), as well as fusiform (res = -.22) and entorhinal gyrus (res = -.17).

Network analysis indicated that ASD showed a stronger negative association with BD, suggesting that BD is a bridge node between ASD and the other diseases. Interestingly, after controlling for the other diseases, ASD exhibited a positive correlation with SCZ in contrast with the PCA and Opel *et al.* ^2^ results. ADHD had a weaker partial correlation with the other nodes that were linked by MDD. SCZ, BD, and OCD maintained the pattern of strong positive associations ( Figure 2D). Centrality measures of the network indicated that SCZ had the highest strength (direct connections to other diseases), BD had the highest closeness (indirect connection to other diseases). BD and MDD had the highest betweenness (the average path between two other diseases), ^5^ see Figure 2E.

## Discussion and conclusion

Our approach suggests that the brain abnormalities found in ASD carry more similarities with MDD, BD, SCZ, and OCD than demonstrated by the original three-component solution in Opel *et al.* ^2^ Indeed, the well-observed pattern of co-occurrence and clinical overlap among ASD and other psychiatric disorders indicates that they share important pathogenic brain mechanisms and risk factors. ^12^^,^^13^ The small association of ASD and the specificity of ADHD abnormalities might be explained by the fact that these are neurodevelopmental disorders, although recent work has supported the idea that the etiology of BD and SCZ also involves a substantial neurodevelopmental basis. ^14^^,^^15^ An alternative and interesting view argue that these disorders could be conceptualized as a neurodevelopmental continuum, in which the symptoms would reflect the severity, timing, and pattern of brain abnormalities, as well as the modulatory effects of genetic and environmental factors. ^14^ The results we obtained here seem to partially reflect Owen and O’Donavan’s ^14^ theoretical understanding of this phenomenon.

To summarize, Opel *et al.* ^2^ advance our understanding of brain morphometric features in highly debilitating psychiatric conditions. Notably, different component solutions can lead to different conclusions, but both approaches were categorical in demonstrating the strong alterations of the hippocampus, fusiform gyrus, pallidum, and putamen. These findings may be of special interest given that the overlapped pattern of symptoms among the major psychiatric diseases usually makes it difficult to accurately diagnose and to prescribe tailored treatment. Moreover, the network approach might help to understand specific disease domains of psychopathology. With more neuroimaging studies of psychiatric disorders becoming available, investigations via shared variation and network perspectives are promising venues for understanding the subtypes of shared pathophysiological patterns.

## Data availability:

### Underlying data

The underlying data of the present study is based on Opel *et al.* ^2^ work and are available on https://doi.org/10.1016/j.biopsych.2020.04.027. The dataset was obtained by copying the effect sizes estimates reported by Opel *et al* ^2^ on Supplementary Table S2 of their manuscript. ^2^ The full manuscript was accessed via the McGill Library Portal ( https://www.mcgill.ca/library/) on September 19, 2020.

© 2020 Society of Biological Psychiatry.
