# Supplementary material for: Brain structural abnormalities in six major psychiatric disorders: shared variation and network perspectives
Source: F1000Res. 2022 Jun 17;10:356. Originally published 2021 May 7. [Version 2] doi: 10.12688/f1000research.51475.2 (PMC10102712; doi:10.12688/f1000research.51475.2)
Supplement: Peer review history

# Peer review: Reviewer response for version 2

**DOI:** 10.5256/f1000research.135143.r160849

**Published:** 2023-04-13

**Authors:** Y. C. Janardhan Reddy, Srinivas Balachander

**Affiliations:** 1 National Institute of Mental Health and Neuro Sciences (NIMHANS), Bengaluru, India; 2 National Institute of Mental Health and Neuro Sciences (NIMHANS), Bangalore, Karnataka, India

This paper presents a re-analysis of a previous cross-disorder ENIGMA paper, to investigate shared or distinct structural brain measures in SCZ, BD, MDD, OCD, ADHD, and ASD. 

 The main message of the paper is that a slightly different statistical approach, which might have certain merits/demerits over the original paper’s (Opel *et al. *(2020)) approach, can lead to significantly different conclusions. 

 The paper raises an important issue, and demonstrates how analyses can be performed differently, but we couldn’t really “see” how different their findings are. The main findings of the regional abnormalities that are shared between disorders (hippocampus, fusiform gyrus and certain sub-cortical regions) are the same as in the original paper (Opel *et al. *(2020)). Also, if the ASD and ADHD groups loaded negatively on Factor 1, can the authors state, with confidence, that the same regional abnormalities are shared within ASD/ADHD as well? We assume that the answer is no.

 While the authors have extensively discussed the “statistical” appropriateness, and advantages of the unidimensional solution, we feel that one should also take clinical relevance and meaningfulness into consideration while choosing the number of components.

 Some of the possible confounds, as to why the 4 psychiatric disorders (SCZ, BD, MDD, OCD) may have loaded together - these were only discussed in passing by the original authors (Opel *et al. *(2020)):

- Shared controls between the samples at various sites (especially between SCZ, BD and OCD and between the ASD and ADHD groups)
- Medication use in the adult psychiatric disorder cohorts - the original ENIGMA papers had also reported results in medication-naive sub-samples (all disorders except BD). These indicated that medication status had a very significant impact on the brain morphometry
- Comorbidity within original samples, that was not accounted for in the main analysis (OCD with comorbid MDD, schizo-affective disorder, etc)

While the authors’ objective was purely to look at statistical considerations, the authors could also suggest how some of the above clinical factors could be taken into account, in future directions.

Is the work clearly and accurately presented and does it cite the current literature?

Yes

If applicable, is the statistical analysis and its interpretation appropriate?

Yes

Are all the source data underlying the results available to ensure full reproducibility?

Yes

Is the study design appropriate and is the work technically sound?

Yes

Are the conclusions drawn adequately supported by the results?

Partly

Are sufficient details of methods and analysis provided to allow replication by others?

Yes

Reviewer Expertise:

I am a psychiatrist working on obsessive-compulsive disorder (also my co-reviewer). We are not experts in statistical analysis, therefore a statistical review may be required.

We confirm that we have read this submission and believe that we have an appropriate level of expertise to confirm that it is of an acceptable scientific standard, however we have significant reservations, as outlined above.

# Peer review: Reviewer response for version 2

**DOI:** 10.5256/f1000research.135143.r160851

**Published:** 2023-02-21

**Authors:** Esther Walton

**Affiliations:** 1 University of Bath, Bath, UK

Filho and colleagues reanalysed data published by Opel *et al.* to characterize the shared (vs unique) variation in brain structural alterations across six psychiatric disorders. In contrast to Opel who reported a 3-factor solution, the authors of this manuscript identify a unidimensional solution. And unlike Opel who reported variation shared across SCZ, BD, OCD and MDD but a unique loading for ASD and ADHD, the current manuscript suggests that there might be a somewhat larger degree of shared variation among all six disorders.

 The manuscript presents an interesting exploration of how analysis decisions can impact results / interpretation of findings, and through that is also a valuable contribution to discussions around ‘researcher degrees of freedom’.

 I have the following comments:

- I suggest citing or reporting in a table the original studies and not just Opel *et al.*, as it is difficult to understand what data the current manuscript / results are based on.
- I assume there is no sample overlap between the original studies (e.g., shared set of controls), which could explain some of the clustering. Might be good to just briefly mention.
- Why is the data only available upon request?
- Why was the data analysed first in R, then SPSS, and then back in R? Wasn’t it possible to analyse everything in one workflow / software? Also, I suggest sharing the R code and / or SPSS syntax.
- It was not fully clear to me whether the only difference between Opel and the current work was simply the 1- vs 3-factor solutions. Were all other methods / parameters the same?
- I’m not fully convinced that the results between the two studies are indeed so different. Figure 2B does show a one-factor solution, but it’s still quite clear that ASD and ADHD load differently onto this factor compared to the other disorders (i.e. negative or not at all versus positive). This seems to align quite closely with the conclusions made by Opel and colleagues. Can the authors comment on that?
- I’m also not fully convinced that either the one or the other method is superior. The third component (Figure 1) was just minimally below the suggested cutoff of 1 (0.98) and issues around using binary cutoffs are discussed at length in the literature (often in the context of p-values or diagnostic criteria). So, a 3-factor solution is not too far-fetched, in the same way that a one-factor solution can also work with this data. In fact, the first factor in Opel seems to explain the same amount of variance as the one factor presented here (~48%), suggesting convergent findings. Can the authors make it clearer whether they believe that a 3-factor solution is in fact misleading? Also, is it possible to present model fit estimates (like RMSEA, CFI, TLI) for both the one- and three-factor solutions?
- What is a " *stronger shared increase*" / " *shared reduction*"? Stronger compared to what? An explanation on how to interpret these findings would be very helpful.

Is the work clearly and accurately presented and does it cite the current literature?

Partly

If applicable, is the statistical analysis and its interpretation appropriate?

Yes

Are all the source data underlying the results available to ensure full reproducibility?

Partly

Is the study design appropriate and is the work technically sound?

Yes

Are the conclusions drawn adequately supported by the results?

Partly

Are sufficient details of methods and analysis provided to allow replication by others?

Partly

Reviewer Expertise:

Structural brain imaging in psychiatry, multi-morbidity / shared signatures across disorders

I confirm that I have read this submission and believe that I have an appropriate level of expertise to confirm that it is of an acceptable scientific standard, however I have significant reservations, as outlined above.

# Peer review: Reviewer response for version 2

**DOI:** 10.5256/f1000research.135143.r141125

**Published:** 2022-06-21

**Authors:** Takahiro Osada

**Affiliations:** 1 Department of Neurophysiology, Juntendo University School of Medicine, Tokyo, Japan

The authors have satisfactorily revised the manuscript by addressing the comments raised in the earlier review.

Is the work clearly and accurately presented and does it cite the current literature?

Yes

If applicable, is the statistical analysis and its interpretation appropriate?

Partly

Are all the source data underlying the results available to ensure full reproducibility?

Yes

Is the study design appropriate and is the work technically sound?

Partly

Are the conclusions drawn adequately supported by the results?

Partly

Are sufficient details of methods and analysis provided to allow replication by others?

Yes

Reviewer Expertise:

neuroimaging

I confirm that I have read this submission and believe that I have an appropriate level of expertise to confirm that it is of an acceptable scientific standard.

# Peer review: Reviewer response for version 1

**DOI:** 10.5256/f1000research.54649.r139204

**Published:** 2022-06-06

**Authors:** Takahiro Osada

**Affiliations:** 1 Department of Neurophysiology, Juntendo University School of Medicine, Tokyo, Japan

The manuscript by de Mendonça Filho *et al*. reanalyzed the data presented in Opel *et al.* to investigate the common brain abnormalities for various psychiatric disorders. Specifically, the authors reconsidered the number of factors in the principal component analysis and demonstrated that a solution of one factor was appropriate while the original study adopted three factors. Based on the unidimensional solution, brain abnormalities in ASD showed a negative component loading, whereas those in MDD, BD, SCZ, and OCD showed positive component loadings. The authors concluded that the same component could explain ASD as well as MDD, BD, SCZ, and MDD. Understanding brain abnormalities related to psychiatric disorders is clinically important, and using a large database of the ENIGA consortium is a plausible way for investigation. However, I have some concerns with the analyses and interpretations of the results in this manuscript.

 Major comments:

1. The authors compared the number of components in the principal component analysis solely based on eigenvalues (Figure 1). I wonder whether the unidimensional analysis explained sufficiently the variance of brain regional effect sizes. According to the original study by Opel *et al.*, the three factors explained 85.14 % of the variance in total. To what extent did this unimodal solution could explain the variance?
2. In the unidimensional analysis, ASD showed a negative component loading while MDD, BD, SCN, and OCD showed positive component loadings. Based on this finding, the authors stated in the Abstract and Discussion that brain abnormalities in ASD had more similarities with the four disorders than the original study. The authors should describe this conclusion with caution. The pattern of brain abnormalities in ASD was not “similar”, but rather opposite to that in the four disorders because of the negative and positive values. The same component could explain ASD and the four disorders rather than the two distinct factors suggested in the original study.
3. The authors calculated the partial correlations among the residuals and conducted network analyses (Figures 2D and 2E). I wonder whether these analyses were valid. It would be reasonable to examine the relationships between the residuals after the component with the same tendency was extracted (i.e., the same sign and nearly the same amount of loading). However, the authors compared the residuals between the disorders for negative loadings (ASD and ADHD) and  positive loadings (MDD, BD, SCN, and OCD). The network analysis seems interesting, but what is the rationale for examining the residuals from data with different tendencies?

Minor comments:

1. Figure 2A was not cited in the text, and how was the “predicted component score” calculated?
2. For evaluating component loadings, why was “λ” used rather than “r”? Is this different from a simple correlation coefficient?
3. It would be helpful to provide a figure for the regional component score map.
4. In the third paragraph in the Results section, the authors stated, “Regional specificities for MDD, BD, SCZ, and OCD were similar to Opel *et al.* results”. Could you elaborate on this?
5. In the fourth paragraph in the Results section, the authors stated, “BD is a bridge node between ASD and the other disease”. Please elaborate on this.
6. Figure 2B: it would be helpful to explain the numbers in the figure legend. I assume these are component loadings.
7. Figure 2C and 2D: the color coding is somewhat counterintuitive. In Figure 2A, red means represents while blue represents negative. In Figure 2D, it would be helpful to add a color scale bar.

Is the work clearly and accurately presented and does it cite the current literature?

Yes

If applicable, is the statistical analysis and its interpretation appropriate?

Partly

Are all the source data underlying the results available to ensure full reproducibility?

Yes

Is the study design appropriate and is the work technically sound?

Partly

Are the conclusions drawn adequately supported by the results?

Partly

Are sufficient details of methods and analysis provided to allow replication by others?

Yes

Reviewer Expertise:

neuroimaging

I confirm that I have read this submission and believe that I have an appropriate level of expertise to confirm that it is of an acceptable scientific standard, however I have significant reservations, as outlined above.

# Response

**Published:** 2022-06-14

**Authors:** Euclides Jose de Mendonca Filho

**Affiliations:** McGill University, Canada

We thank the reviewer for the thorough revision of our manuscript. Bellow, we address the comments to improve readers’ understanding of our findings.

***Major comments:***

***1. The authors compared the number of components in the principal component analysis solely based on eigenvalues (Figure 1). I wonder whether the unidimensional analysis explained sufficiently the variance of brain regional effect sizes. According to the original study by Opel et al., the three factors explained 85.14% of the variance in total. To what extent did this unimodal solution could explain the variance?***

 Although the unimodal solution explains less of the variance, the consideration of a unidimensional solution was based on three criteria:1) the scree plot of eigenvalues, 2) the exhaustive parallel analysis procedure that identified one component as a recurrent robust solution, and 3) the inspection of the Opel *et al.* solution that indicated that the second and third components were defined by only one variable each. The inspection of the original 3-factors solution was the motivator for the reanalysis presented in our manuscript. Since principal component analysis is a data reduction technique, considering components with only one variable seemed redundant, and an indication that the number of factors extracted should be reconsidered. We added the explained variance in the text.

 Now it reads:

 "The unidimensional solution explained 48.3% percent of the six-brain structural alterations and indicated that the effect size of the differences in brain structure between ASD patients and controls shared a significant negative component loading (λ = -0.30, *p* = 0.04) with brain abnormalities of the remaining five diseases (Figure 2). Although the unimodal solution explains less of the variance than the original work (the three-factor solution explained 85.14% of variance), the consideration of a unidimensional solution was based on several criteria. Namely, the scree plot of eigenvalues, the exhaustive parallel analysis procedure that identified one component as a recurrent robust solution, and the inspection of the Opel *et al*. ^2 ^solution that indicated additional second and third components defined by only one variable each. Since principal component analysis is a data reduction technique, considering components with only one variable seemed redundant, and an indication that the number of factors extracted should be reconsidered."

***2. In the unidimensional analysis, ASD showed a negative component loading while MDD, BD, SCN, and OCD showed positive component loadings. Based on this finding, the authors stated in the Abstract and Discussion that brain abnormalities in ASD had more similarities with the four disorders than the original study. The authors should describe this conclusion with caution. The pattern of brain abnormalities in ASD was not “similar”, but rather opposite to that in the four disorders because of the negative and positive values. The same component could explain ASD and the four disorders rather than the two distinct factors suggested in the original study.***

 We agree with the reviewer that the observed pattern is not similar but is negatively associated with alterations in SCZ, BD, MDD, and OCD. We clarified this information throughout the manuscript.

***3. The authors calculated the partial correlations among the residuals and conducted network analyses (Figures 2D and 2E). I wonder whether these analyses were valid. It would be reasonable to examine the relationships between the residuals after the component with the same tendency was extracted (i.e., the same sign and nearly the same amount of loading). However, the authors compared the residuals between the disorders for negative loadings (ASD and ADHD) and positive loadings (MDD, BD, SCN, and OCD). The network analysis seems interesting, but what is the rationale for examining the residuals from data with different tendencies?***

 Our framework is based on the RDoC initiative that acknowledges that common brain abnormalities can potentially explain psychiatry comorbidities. We aimed at investigating patterns of shared variance independently of the direction of observed brain alterations. PCA and Network analysis are both appropriate for modelling opposite tendencies, and the use of network analysis is aimed at further interrogating the relationship between brain alterations by investigating the function of the observables in the network. This allowed us to obtain information about which observables are dominant in the network in terms of the strength of relations with other observables, for instance.

***Minor comments:***

***Figure 2A was not cited in the text, and how was the “predicted component score” calculated?***

 Thank you for bringing this to our attention, we added Figure2A to the 11th paragraph.

 We also added information about the estimation of the component score.

 Now it reads:

 "The principal components algorithm was used for dimensional extraction, and component scores were obtained using the regression weights (Thurstone method), which are obtained by multiplying the inverse of the observed variable correlation matrix by the matrix of factor loadings."

***1. For evaluating component loadings, why was “λ” used rather than “r”? Is this different from a simple correlation coefficient?***

 The λ symbol represents a factor or component loading, thus it is not a Pearson correlation coefficient, although λ reported are standardized yielding a similar interpretation to a simple correlation coefficient. 

***2. It would be helpful to provide a figure for the regional component score map.***

 This information is depicted in Figure 2A, we added a sentence to clarify this. 

***3. In the third paragraph in the Results section, the authors stated, “Regional specificities for MDD, BD, SCZ, and OCD were similar to Opel et al. results”. Could you elaborate on this?***

 Thank you for bringing this to our attention. The sentence now reads:

 "Regional specificities for MDD, BD, SCZ, and OCD were similar to Opel *et al*. results, in which large absolute residuals were found in the rostral ACC and the medial orbito frontal cortex (OFC) for MDD, parahippocampal gyrus and pallidum for BD and superior temporal gyrus and medial OFC for SCZ."

***4. In the fourth paragraph in the Results section, the authors stated, “BD is a bridge node between ASD and the other disease”. Please elaborate on this.***

 Thank you for bringing this to our attention. The sentence now reads:

 "Network analysis indicated that ASD showed a stronger negative association with BD, suggesting that BD is a bridge node between ASD and the other diseases. Thus, the brain alterations in BD may connect ASD alterations to the other nodes, and if the BD node is removed, the observed shared variance with SCZ, OCD, and MDD is likely to decrease"

***5. Figure 2B: it would be helpful to explain the numbers in the figure legend. I assume these are component loadings.***

 The reviewer is correct, we added a sentence to grant a better understanding of Figure 2A

***6. Figure 2C and 2D: the color coding is somewhat counterintuitive. In Figure 2A, red means represents while blue represents negative. In Figure 2D, it would be helpful to add a color scale bar.***

 Thank you for your suggestion, we altered Figures 2C and 2D for clarity.
